# Nanoparticles of *Costus* *speciosus* Ameliorate Diabetes-Induced Structural Changes in Rat Prostate through Mediating the Pro-Inflammatory Cytokines IL 6, IL1β and TNF-α

**DOI:** 10.3390/molecules27031027

**Published:** 2022-02-02

**Authors:** Duaa Bakhshwin, Khadija Abdul Jalil Faddladdeen, Soad Shaker Ali, Samar Mohammed Alsaggaf, Nasra Naeim Ayuob

**Affiliations:** 1 Department of Pharmacology, Faculty of Medicine, King Abdulaziz University, Jeddah 22254, Saudi Arabia; dbakhshwin@kau.edu.sa; 2 Biology Department, Faculty of Science, King Abdulaziz University, Jeddah 22254, Saudi Arabia; Kfaddladdeen@kau.edu.sa; 3 Department of Anatomy, Faculty of Medicine, King Abdulaziz University, Jeddah 22254, Saudi Arabia; soadshaker@gmail.com (S.S.A.); Salsaggaf@gmail.com (S.M.A.); 4 Yousef Abdul Latif Jameel Scientific Chair of Prophetic Medicine Application, Faculty of Medicine, King Abdulaziz University, Jeddah 22254, Saudi Arabia; 5 Department of Histology and Cell Biology, Faculty of Medicine, Assuit University, Assuit 98467, Egypt; 6 Department of Medical Histology and Cell Biology, Faculty of Medicine, Damietta University, Damietta 34511, Egypt

**Keywords:** diabetes mellitus, inflammation, cytokines, *Costus* *speciosus*, nanoparticles, prostate, testosterone, Bcl-2, Ki67, insulin

## Abstract

Diabetes mellitus is a common global health problem. Among the complications that are frequently associated with DM are the alternation of sexual function and fertility, especially in young men. This study aimed to assess the efficacy of nanoparticles of *Costus speciosus* (*C. speciosus*) in preserving the prostatic structure of diabetic rats and to explore the mechanism behind this effect. A model of DM was induced in male albino rats by a single intraperitoneally injection of streptozotocin (STZ, 60 mg/kg body weight). Five groups (*n* = 10 each) of rats were included in this study: the control, *C. speciosus* gold nanoparticles-treated (150 mg/kg body weight through gastric intubation for 30 days), untreated diabetic, metformin-treated diabetic (500 mg/kg/day gastric intubation for 30 days) and the *C. speciosus*-treated diabetic group. The blood glucose, insulin and testosterone levels as well as oxidants/antioxidants status were assessed in the serum. Gene expression of proinflammatory cytokines TNF-α, IL1β and IL-6 were assessed in the prostate homogenate. At the end of the experiment, the rats were sacrificed and the prostate was dissected out and prepared for histopathological and immunohistochemistry study using Ki67 and Bcl-2. *C. Speciosus* nanoparticles significantly decreased (*p* = 0.03) the blood glucose level while significantly increasing insulin (*p* = 0.01) and testosterone (*p* = 0.04) levels compared to the untreated diabetic rats. Oxidants/antioxidants status was markedly improved after administration of *C. speciosus.* Prostatic expression of the mRNA of pro-inflammatory cytokines IL-6, IL1β and TNF-α was down-regulated in metformin- and *C. speciosus*-treated rats. The histological structure of the ventral prostate was preserved in metformin- and *C. speciosus*-treated diabetic rats with a significantly thicker epithelial cell layer and significant increase immunoexpression in Bcl-2 and Ki67. In conclusion, the protective effect induced by *C. speciosus* nanoparticles on the prostate of diabetic rats might be directly mediated through the down-regulation of inflammatory cytokines and the up-regulation of antioxidant activity and indirectly mediated through the anti-hyperglycemic effect through enhancing insulin secretion.

## 1. Introduction

Type 2 diabetes mellitus (DM) has become “a modern-day plague” by reaching epidemic levels throughout the world. The prevalence of type 2 DM (3.9–18.3%) in the Arab Gulf countries is amongst the highest prevalence internationally, and is still rising quickly. Unfortunately, DM is frequently associated with serious complications such as nephropathy, neuropathy, retinopathy and male infertility [1,2]. Oxidative stress, induced by a high glucose concentration in blood and tissue fluids, was reported to induce male reproductive complications [3]. It was found that antioxidant treatment, either from natural or synthetic sources, recovers the glycemic index and prevents oxidative stress resulting from free radicals, therefore minimizing the occurrence of diabetic complications [4]. Several studies have reported a direct relationship between diabetes and prostate pathologies which may be a consequence of metabolic abnormalities and changes in sex hormone levels in diabetic patients [5].

The number of people who use plant-based remedies as alternative or complementary medicine is increasing [6]. This highlights the need for more rigorous research to explore and test the efficacy of plants and natural products with safe antidiabetic activity in order to validate these substances in the management of DM specifically in young males. *Costus speciosus* (Koen ex. Retz.) Sm. (syn. *Cheilocostus speciosus*, Family: Costaceae) is an ethnic antidiabetic plant which is popularly known by the name ‘insulin plant’ and also ‘crepe ginger’ [7]. *C. speciosus* was among the most effective Islamic traditional medicinal (ITM) plants used for the management of ‘khadar’ or ‘paresthesia’, a common sensory symptom of multiple sclerosis and peripheral neuropathies [8].

Nanoparticles (NPs) are currently used to improve therapeutic activity of the natural product. “Lipid-based nanoparticles, which include solid lipid nanoparticles (SLN) and nanostructured lipid carriers (NLCs)” are considered promising drug delivery system for lipophilic pharmaceuticals. Being formed of both solid and liquid lipids, this improves the “drug loading efficiency and stability” of NLCs. NLCs were developed to overcome the problem of drug expulsion from SLNs upon storage due to crystallization and phase transition of solid lipids [9]. Plant extracts provide both reducing and stabilizing agents for the formation of nanoparticles concurrently with inducing their medicinal values to the particles, which enhance biomedical applications of nanoparticles [10].

Based on recent scientific reports, it has been observed that commonly used methods have helped to design more effective nanomaterials while keeping the hazards of the substances at a minimum. In nanomedicine, the significance of nanotoxicology is particularly important to avoid the toxicity of drug nanocarriers [11]. Recently, Zhu et al. reported that nanotechnology will become more widely involved in the diagnosis and treatment of diseases in the future, potentially helping to overcome bottlenecks under existing medical methods [12].

Although the antidiabetic effect of *C. speciosus* [6,13] and its nanoparticles [14,15] was previously reported, the impact of *C. speciosus* nanoparticles on diabetic-induced prostatic changes was not sufficiently investigated. Therefore, this study aimed primarily to fill this knowledge gap as it was designed to assess the efficacy of *C. speciosus* nanoparticles in preserving the prostatic structure in a streptozotocin (STZ)-induced animal model of DM, controlling the blood glucose level, and exploring the mechanism behind this effect.

## 2. Materials and Methods

### 2.1. Preparation of C. speciosus Extract (CSE)

Compritol 888 ATO (Glycerol dibehnate), Precirol ATO5 (Glycerol distearate), were generously provided as a free sample by Gattefosse Company, France. Pluronic F-68 was purchased from Sigma Chemicals, St. Louis, MO, USA. Oleic acid (Alpha Chemicals Co., Cairo, Egypt), Tween^®^80 (Adwic, El-Naser chemical co., Cairo, Egypt) were utilized in this study. Other chemicals and reagents were of analytical grades.

*C. speciosus* was obtained from the herbal market in Saudi Arabia and was be verified by the Botanist at the Faculty of Sciences, King Abdulaziz University. 

*C. speciosus* was washed with water, dried, cut into pieces and powdered in a mechanical grinder as was previously described in [16]. The powder of the plant (10 g) was extracted with de-ionized (100 mL) water. The mixture was stirred at 70 °C and 200 rpm then filtered by using filter paper. After filtration, the resulting aqueous extract was then lyophilized using a freeze dryer and stored in a desiccator until further use. The dry extract yield was approximately 10% of the crude material. The extract was prepared at a concentration of 10 mg/mL 50% ethanol solution.

### 2.2. Preparation of C. speciosus Extract-Loaded Nanostructured Lipid Carriers (CSE-NLCs)

*C. speciosus* extract was loaded within NLCs using the “emulsification–solvent evaporation technique followed by ultrasonication” with minor modifications [17]. In brief, the lipid phase was prepared by dissolving dried *Costus speciosus* extract and 500 mg of a solid lipid mixture (Compritol^®^888 ATO (150 mg) and Precirol^®^ATO5 (350 mg) and liquid lipid (oleic acid 500 mg) in 2 mL of ethanol (1:1 *v*/*v*) at 70 °C. The aqueous phase of 20 mL distilled water in the presence of PluronicF-68/Tween^®^80 mixture (1:1) as a stabilizer was heated to the same temperature. The aqueous phase was then added to the lipid phase under stirring at 2000 rpm and 70 °C, and then mixed for 15 min. The resulting pre-emulsion was sonicated by probe-type sonicator (Cole-Parmer, Vernon Hills, IL, USA) for 10 min at pulse ON for 3 s and pulse OFF for 5 s (40 W). The obtained dispersion was cooled under stirring at 1000 rpm for 1 h to obtain the aqueous NLC dispersions. As a control, CSE free plain NLCs were prepared using the same protocol. Table 1 shows the composition of CSE-NLCs formulation.

### 2.3. Characterization of CSE-NLCs

#### 2.3.1. Particle Size and Zeta-Potential Measurements

The particle size distribution and polydispersity index (PDI) of CSE-loaded NLCs were measured at 25 °C by the dynamic laser light scattering (DLS) technique using “Zetasizer Nano ZS (Malvern Instruments, Worcestershire, UK) equipped with a backscattered light detector operating” at 173°. The zeta-potential of NLCs dispersion was determined by laser Doppler anemometry using a Malvern Zetasizer Nanoseries ZS. The measurements were performed in triplicate.

#### 2.3.2. Encapsulation Efficiency (EE) and Loading Capacity (DL)

The EE% of CSE within NLCs was indirectly estimated by assessing free unloaded spectrophotometrically using (Jenway Model 6305, U.K) at λ max = 372 nm, after separation via cooling centrifugation at 4 °C, 14,000 rpm, for 60 min, using a (bench-top refrigerated centrifuge Centurion Scientific Ltd., W. Sussex, UK) [18]. EE (%) was calculated according to the following equation:

(1) $$EE\left(\%\right)=\frac{\left(T-C\right)}{T}\times \;100$$

where *T* is the total amount of extract added, and *C* is the amount of free unentrapped extract in the supernatant. Each experiment was performed in triplicate.

On the other hand, the solution of CSE loaded nanostructured lipid carriers was dried and weighed. The *DL*% of loaded CSE nanostructured lipid carriers nanoparticles was determined according to the following equation:

(2) $$DL\left(\%\right)=\frac{\left(T-C\right)}{\text{Total material weight}}\times \;100$$

#### 2.3.3. The Particle Morphology Using Scanning Electron Microscopy 

The surface morphology of CSE-NLCs was visualized using scanning electron microscopy (Jeol, JSM-5200, Tokyo, Japan). The sample of CSE-NLCs was prepared by placing a droplet on an aluminum specimen stub then left to dry overnight, and finally the sputter was coated with gold prior to imaging. An acceleration voltage of 15 kV was utilized in SEM.

### 2.4. Animals and Experimental Design

The Biomedical Research Ethics Committee, Faculty of Medicine, King Abdulaziz University approved this study (18-2021) which was conducted on fifty albino male rats (200–240 g). They were purchased from the animal house at King Fahed Medical Research Center, King Abdulaziz University, Jeddah, KSA. The control group (*n* = 10) received 1 mL of saline through gastric intubation for 30 days. *C. speciosus*-treated control group received gold nanoparticles containing *C. speciosus* at a dose of 150 mg/kg body weight (bw) through gastric intubation for 30 days as was described by [19].

**Figure 1:**
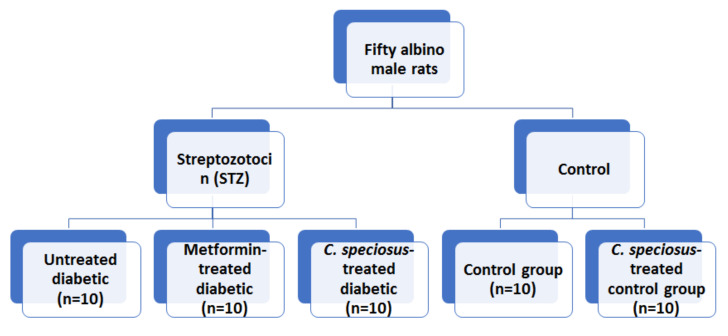
Graphical scheme of the study design.

A model of type 2 DM was induced in thirty rats using intraperitoneal injection of freshly prepared streptozotocin (STZ, Sigma Aldrich Chemical Company, CO., St. Louis, MO, USA) in a single dose (60 mg/kg bw) of intraperitoneal injection according to [20]. Blood glucose for all rats will be checked after three days to confirm diabetes. A blood glucose level ≥ 250 mg/dL was considered the cut-off point of the rats to be diabetic [21]. Diabetic rats were divided into 3 groups (*n* = 10 each): untreated diabetic, metformin-treated diabetic and *C. speciosus*-treated diabetic. Metformin hydrochloride (Chemical Industrial Development Co., Ltd., El Omraniya, Egypt) was used for pharmacological validation of *C. speciosus* (500 mg/kg/day for 30 days) through gastric intubation as was previously described by [22]. Figure 1 showed a graphic scheme of the study design.

### 2.5. Biochemical Assessment

Blood was collected from the orbital venous plexus of the rat, then centrifuged for 15 min in order to separate the serum and stored at −80 °C. The blood glucose level (BGL) was measured using enzymatic glucose kits (Merck KGaA, Darmstadt, Germany), while serum insulin level was assessed using insulin ELISA kits (Merck KGaA, Darmstadt, Germany) according to [21].

The malonaldehyde (MDA) level (Biodiagnostic; Dokki, Egypt) was assessed spectrophotometrically according to [23] using the Thiobarbituric Acid Reactive Substances (TBARS) Assay Kit. 

The level of Superoxide dismutase (SOD) was measured using an assay kit (Biodiagnostic; Egypt) according to the method described by [24]. Glutathione peroxidase (GPX) (Randox Labs, Crumlin, UK) and catalase (CAT) (Biodiagnostic; Egypt) were assessed according to the method of [23]. Testosterone was assessed in the serum according to the method described by [25]

### 2.6. Assessment of Gene Expression Using Quantitative Real-Time Polymerase Reaction (qRT-PCR)

RNA was extracted from the prostatic tissue samples using TriFast^™^ reagent (PeqLab, Erlingen, Germany, Cat No.: 30-2010) then the extracted RNA was reverse transcribed using a SensiFAST™ cDNA Synthesis Kit for qRT-PCR (Bioline USA Inc., Memphis, TN, USA, Cat No.: BIO-65053), according to the manufacturer’s instruction, and the resulting cDNA was stored at −80 °C.

The qRT-PCR reactions were performed using SensiFAST^TM^ SYBR Lo-ROX Kit (Bioline USA Inc., Memphis, USA, Cat No.: BIO-94002) on the Applied Biosystems 7500 real-time PCR detection system (Life Technology, San Francisco, USA). Gene-specific primers utilized in this study were shown in Supplementary Table S1. They were designed through the Primer3 software (v.0.4.0) and the NCBI/Primer-BLAST program was utilized to check its specificity. The primers were purchased from Willowfort™.

The PCR mixture was prepared as was described in a previous study [26]. A negative control reaction containing no template was run in each experiment. The melting curve was analyzed in order to document PCR products’ specificity. The relative quantification for each gene expression in the tissue samples was calculated using comparative threshold (ΔΔCt) method with the GAPDH as the internal control gene. The fold change was calculated and linearized by the 2^−ΔΔCt^ arithmetic.

### 2.7. Histopathological Assessment

The animals were sacrificed by decapitation 24 h after the last dose of *C. speciosus*. The abdomen was opened, and the prostate was dissected out and then removed. It was fixed in 10% neutral buffered formalin, dehydrated in ascending grades of alcohol, and then embedded in paraffin and processed to obtain 5 micron-thick sections stained by Hematoxyline & Eosin to assess the histopathological changes according to [27]. A set of paraffin sections was immunostained using an avidin–biotin technique Bcl2, (catalog number N-19, sc-492, Santa Cruz Biotechnology, at dilution 1:250), and Ki67 (catalog number (ab15580), Abcam, Cambridge, UK at dilution 1:100).

A light microscope BX-51 (Olympus, Hamburg, Germany) connected to a digital camera was used for photographing. The Pro Plus image analysis software version 6.0 was used for semi-quantitative assessment of immunoexpression of used antibodies. The number of Ki67-positive cells as well as the area percent of Bcl2 was assessed in in non-overlapping five sections in each animal at magnification ×200. Epithelial thickness lining the prostatic acini was measured in non-overlapping five sections in each animal at magnification ×100. Ten readings from each section were taken and the mean for each animal was calculated according to [28].

### 2.8. Statistical Analysis

The raw data obtained from the biochemical and immunohistochemical assessment was analyzed using Statistical Package of Social Science Program (SPSS, SPSS Inc., Chicago, IL, USA) version 20 and the results were presented as mean ± standard deviation (SD). The normality of the data was checked. The parametric data were compared using analysis of variance (ANOVA) followed by Bonferroni *post-hoc* test to avoid multiple-comparison effect. *p* < 0.05 was considered significant.

## 3. Results

The results of this study presented many findings regarding the characterization of the CSE-NLCs and its efficacy on some biochemical parameters included the serum testosterone level, antioxidants profile and gene expression of pro-inflammatory cytokines in the prostate as well as the histopathological changes in the prostate.

### 3.1. Preparation and Characterization of CSE-NLCs

**Figure 2:**
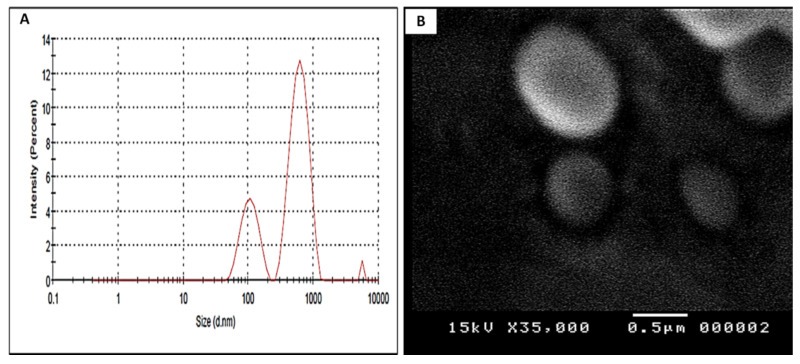
(**A**) Particle size distribution measured by dynamic laser light scattering (DLS) technique. (**B**) Scanning electron micrograph shows uniform and spherical shape of C. speciosus extract-loaded nanostructured lipid carriers (CSE-NLCs) with no evident signs of aggregation.

The CSE-NLCs, at the concentration 3 mg/mL, were performed using emulsification-solvent evaporation technique followed by ultrasonication. Malvern ZetaSizer revealed that the average particle size, PDI, zeta potential, drug loading and encapsulation efficiency of CSE-NLCs were 568.4 ± 0.51 nm, 0.498 ± 0.06, −39 ± 2.3 mV, 4.73 ± 0.05% and 89.20 ± 1.9%, respectively (Figure 2A, Table 2). As depicted by the SEM micrographs (Figure 2B), CSE-NLCs displayed a uniform and spherical shape with no evident sign of aggregation.

### 3.2. Effect of C. speciosus on Body and Prostate Weight

The body weight of the rats showed no significant difference between all groups at the start of the experiment, while those of the untreated diabetic rats showed a significant reduction (*p* = 0.01) in their body weight by the end of the experiment when compared to the control. Although the body weight of metformin- and *C. speciosus*-treated diabetic rats increased at the end of the experiment, this was of no statistical significance (Table 3).

Although the prostate weight was significantly reduced (*p* = 0.02) in untreated diabetic rats compared to that of the control group, it showed insignificant increase in metformin- and *C. speciosus*-treated diabetic rats compared to untreated diabetic rats Table 3.

### 3.3. Effect of C. speciosus on BGL and Insulin

The fasting BGL increased above 250 mg/dl in all rats that received STZ injection after 7 days and remained significantly increased (*p* < 0.001) in untreated diabetic rats compared to the control group till the end of the experiment. On the other hand, the BGL showed a significant decrease in both metformin- and *C. speciosus*-treated diabetic rats (*p* = 0.01 and *p* = 0.03) compared to the untreated diabetic rats, respectively, with no significant difference between the two groups (Table 3).

The serum insulin was significantly reduced (*p* < 0.001) in untreated diabetic rats compared to the control, while it showed a significant increase (*p* = 0.003, *p* = 0.01) in both metformin- and *C. speciosus*-treated diabetic rats (Table 3).

### 3.4. Effect of C. speciosus on Oxidants/Antioxidants Profile

It was found that STZ-induced diabetic rats had a significantly increased (*p* = 0.004) MDA level while treatment with metformin and *C. speciosus* (*p* = 0.04, *p* = 0.01) could significantly reduce it when compared to untreated diabetic group, respectively (Table 3).

On the other hand, STZ-induced DM resulted in a significantly reduced serum level of SOD (*p* < 0.001), GPX (*p* = 0.003) and CAT (*p* < 0.001) when compared to the control. Metformin- and *C. speciosus*-treated diabetic rats showed a significant increase in SOD (*p* = 0.01, *p* = 0.002), GPX (*p* = 0.02, *p* = 0.01) and CAT (*p* = 0.001, *p* < 0.001) levels, respectively, compared to untreated diabetic rats (Table 3).

### 3.5. Effect of C. speciosus on Testosterone Level

STZ-induced DM was associated with a significantly lower (*p* < 0.001) testosterone level, while treatment with metformin and *C. speciosus* (*p* = 0.02, *p* = 0.04) resulted in a significant increase in its level compared to the untreated diabetic rats, respectively (Table 3).

### 3.6. Effect of C. speciosus on Gene Expression of Pro-Inflammatory Cytokines

**Figure 3:**
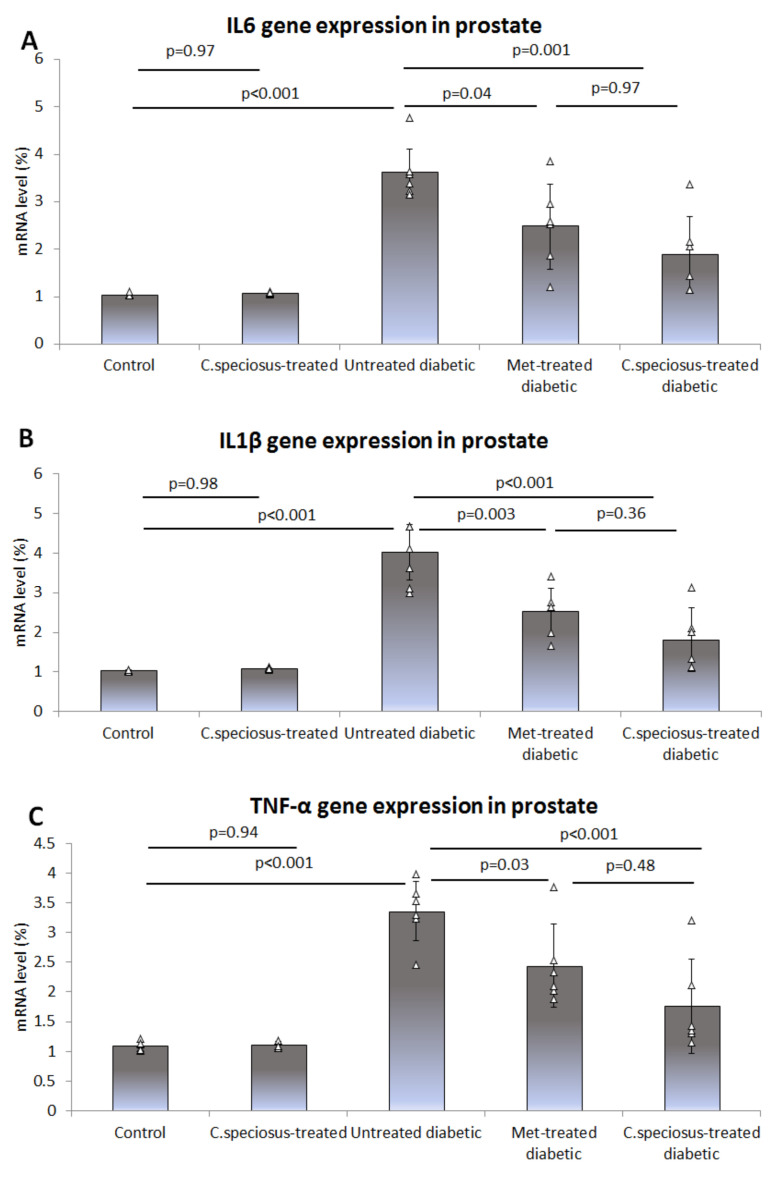
Expression of the mRNA of pro-inflammatory cytokines IL-6 (**A**), IL1β (**B**) and TNF-α (**C**) in the ventral prostate of the studied groups was estimated using qRT-PCR. Results are presented as mean ± SD, *n* = 10. The studied groups were compared using one-way ANOVA test followed by the Bonferroni *post-hoc* test. Significance is considered at *p* < 0.05.

Expression of the mRNA of pro-inflammatory cytokines IL-6, IL1β and TNF-α in the ventral prostate of the studied groups was assessed using qRT-PCR. It was observed that the mRNA of IL-6 was significantly up-regulated in untreated diabetic rats compared to that of the control (3.61 ± 0.59 versus 1.03 ± 0.03, *p* < 0.001), while it was down-regulated in metformin- (2.48 ± 0.91 versus 3.61 ± 0.59, *p* = 0.04) and *C. speciosus*-treated diabetic rats (1.89 ± 0.84, versus 3.61 ± 0.59, *p* = 0.001) when compared to the untreated diabetic rats (Figure 3A).

The expression of IL-1β mRNA in the prostate significantly increased in untreated diabetic rats compared to that of the control (4.03 ± 0.70 versus 1.04 ± 0.02, *p* < 0.001), while it was reduced in metformin- (2.52 ± 0.62 versus 4.03 ± 0.70, *p* = 0.003) and *C. speciosus*-treated diabetic rats (1.81 ± 0.78, versus 4.03 ± 0.70, *p* < 0.001) when compared to the untreated diabetic rats (Figure 3B).

Regarding the expression of the mRNA of TNF-α in the ventral prostate, it was significantly up-regulated in untreated diabetic rats when compared to that of the control (3.36 ± 0.52 versus 1.09 ± 0.08, *p* < 0.001), while it was down-regulated in metformin- (2.44 ± 0.69 versus 3.36 ± 0.52, *p* = 0.03) and *C. speciosus*-treated diabetic rats (1.78 ± 0.78, versus 3.36 ± 0.52, *p* < 0.001) when compared to the untreated diabetic rats (Figure 3C).

### 3.7. Effect of C. speciosus on Histopathological Structure of the Ventral Prostate

**Figure 4:**
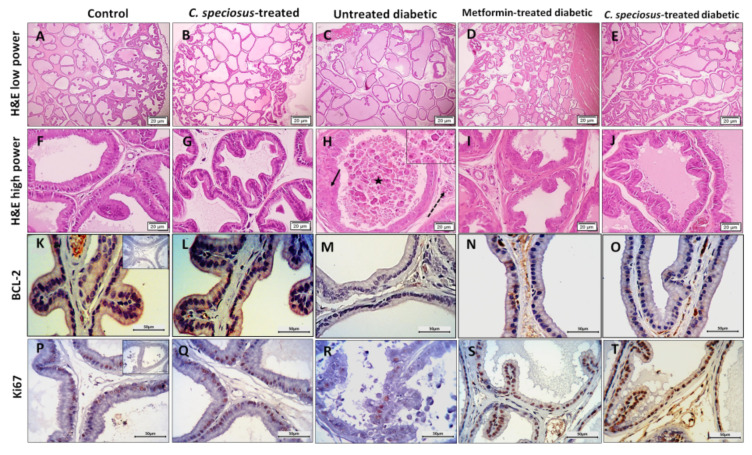
Sections of ventral prostates of control and *C. speciosus-*treated rats show intact prostatic acini with different sizes and shapes and with many epithelial folds while those of untreated diabetic rats show enlarged, widely separated acini with reduced height of acinar epithelium, less epithelial folds and many degenerated epithelial cells (arrow) that appear in the lumen (star) as well as dilated capliraries (interrupted arrow). Ventral prostate of metformin- and *C. speciosus*-treated diabetic rats show much preserved structure (Haematoxylin and eosin stained sections (**A**–**J**). Ki67 and BCL-2 immunoexpression in ventral prostate are shown (**K**–**T**). Inserts of the control group show the negative control of the immunostaining.

**Figure 5:**
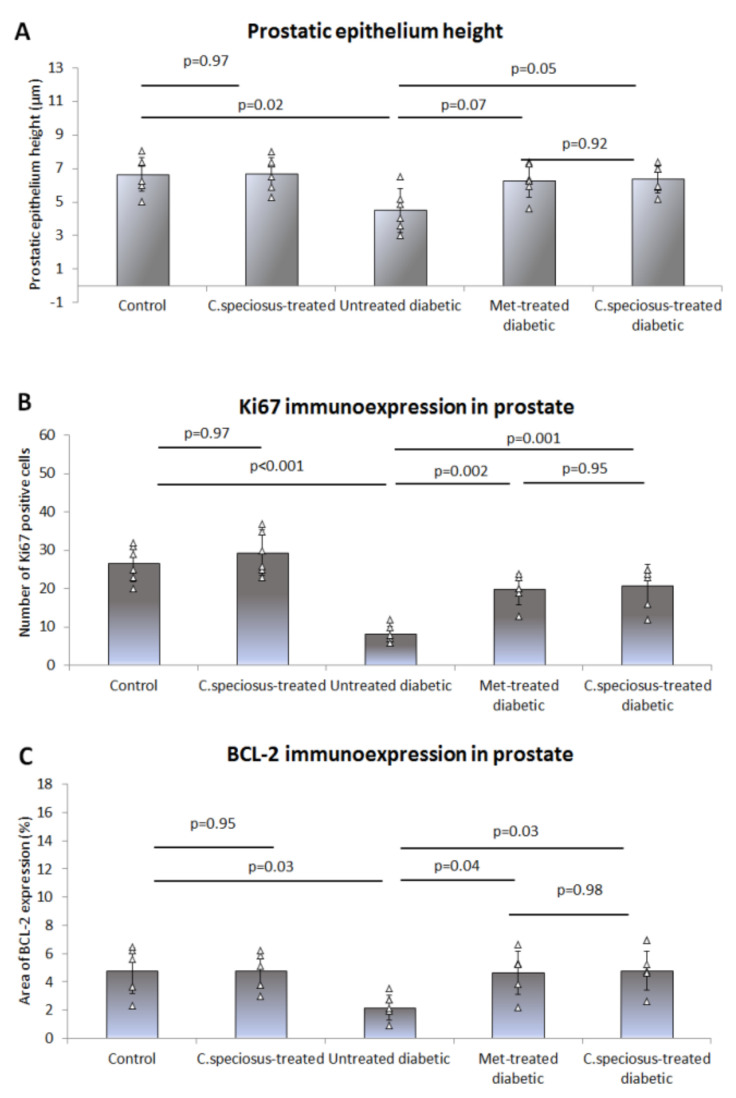
Prostatic epithelium height (**A**), immunoexpression of Ki67 (**B**) and BCL-2 (**C**) were measured in the ventral prostate of the studied groups. Results are presented as mean ± SD, *n* = 10. The studied groups are compared using one-way ANOVA test followed by Bonferroni *post-hoc* test. Significance is considered at *p* < 0.05.

Haematoxylin and eosin-stained sections of the ventral prostate of control rats showed intact crowded prostatic acini with different sizes and shapes surrounded by thin fibromuscular stroma. The epithelium lining the acini was columnar and formed few epithelial folds projecting into the acinar lumen (Figure 4). Prostates of *C. speciosus-*treated rats showed no histological alternations compared to those of the control rats. The ventral prostates of untreated diabetic rats showed enlarged acini that appeared widely separated from each other. The acinar epithelium had significantly reduced thickness (4.49 ± 1.25, versus the control, 6.64 ± 1.11, *p* = 0.02) compared to the control rats with absence of epithelial folds in most of the acini (Figure 5). Some acini showed many degenerated epithelial cells with dark cytoplasm and deeply stained nuclei and were shed into the acinar lumen. Many capillaries were dilated.

Ventral prostate of metformin- and *C. speciosus*-treated diabetic rats appeared with much preserved structure compared to those of the untreated diabetic rats. The acini showed many epithelial folds with thicker epithelial cell layers (6.27 ± 1.01 and 6.35 ± 0.8 versus 4.49 ± 1.25, *p* = 0.07, *p* = 0.05, in metformin- and *C. speciosus*-treated diabetic rats, respectively) when compared to the untreated diabetic rats (Figure 4 and Figure 5). The degenerated epithelial cells were less frequently observed compared to the untreated diabetic group.

Regarding the immunoexpression of Bcl-2 in the ventral prostate, it was noticed that there was a positive Bcl-2 expression in the epithelial cells in both control and *C. speciosus-*treated rats, while those of the untreated diabetic rats showed a significant decrease (2.16 ± 0.9 versus 4.79 ± 1.6, *p* = 0.03) compared to the control. The ventral prostate of metformin- and *C. speciosus*-treated diabetic rats showed a significant increase in Bcl-2 immunoexpression compared to untreated diabetic rats (4.64 ± 1.5 and 4.79 ± 0.8 versus 2.16 ± 0.9, *p* = 0.04, *p* = 0.03, respectively) (Figure 4 and Figure 5).

When it came to Ki67 immunoexpression in the ventral prostate, it was observed that there was a large number of Ki67-positive epithelial cells in both control and *C. speciosus-*treated rats, while those of the untreated diabetic rats showed a significant decrease (8.17 ± 2.4 1 versus 26.66 ± 4.76, *p* < 0.001) when compared to the control (Figure 3 and Figure 4). Prostate of metformin- and *C. speciosus*-treated diabetic rats showed a significant increase in Ki67-positive cells compared to untreated diabetic rats (19.83 ± 3.87 and 20.83 ± 5.52 versus 8.17 ± 2.4, *p* = 0.002, *p* = 0.001, respectively) (Figure 4 and Figure 5).

## 4. Discussion

Fluctuations in blood glucose level, which might occur during the optimum anti-diabetic regimens, as well as DM-associated oxidative stress represent a big challenge in managing DM [29]. Thus, the replenishment of insulin-producing β-cells is considered fundamental in managing DM and preventing its complications [30]. Among the unfavorable effects of DM on male fertility are erectile dysfunction, retrograde ejaculation and reduced levels of testicular hormone and seminal quality [31]. In this study, we examine the anti-hyperglycemic effect of *C. speciosus* in STZ-induced DM and the impact of this effect on the diabetes-induced structural changes in rat prostates in comparison to that of metformin. We hypothesized that *C. speciosus* preserves the prostatic structure against hyperglycemia-induced changes comparable to metformin by down-regulating the expression of pro-inflammatory cytokines and enhancement of the antioxidants.

This study showed that *C. speciosus* significantly reduced STZ-induced hyperglycemia and significantly increased serum insulin in diabetic rats in a comparable level to that of metformin. These findings were in agreement with those previously reported in STZ-induced animal models of DM [13]. They attributed the anti-hyperglycemic effect of *C. speciosus* extract to increased insulin synthesis and release by pancreatic β-cells, increased sensitivity of cell receptors to insulin, and enhanced expression of insulin, insulin receptor A (IRA), glucokinase (GK), succinate dehydrogenase (SDH), pyruvate kinase (PK), and glycogen synthase activity [13]. On the other hand, the *C. speciosus*-induced hypoglycemic effect observed in alloxan-induced DM was associated with increased glycogenesis and decreased gluconeogenesis [32]. The anti-hyperglycemic actions of *C. speciosus* were attributed to eremanthin and costunolide [33]. Regarding the nanoparticles of *C. speciosus,* their glucose-lowering action was attributed to inhibition of hepatic glucose production, promotion of glucose utilization through suppressing the transcription of genes involved in pancreatic insulin and hepatic glucose production and stimulation of the expression of GLUT-4 gene in skeletal muscles of STZ-induced diabetic rats [14].

STZ-induced DM, in this study, was associated with marked increase in MDA, an index of lipid peroxidation, and a significant reduction in GSH, CAT and SOD. This observation might be attributed to increased utilization of these antioxidants under hyperglycemia-induced oxidative stress in pancreatic beta cells [34]. *C. speciosus*-treated diabetic rats, in this study, showed a significant increase in SOD, GPX and CAT levels as well as a significant reduction in MDA. The antioxidant effect of *C. speciosus* extract was previously documented in different animal models of chronic diseases as hyperlipidemia [16], DM [35,36], cancer and heart diseases [37]. The free radical-scavenging ability of *C. speciosus* was attributed to the diverse phytochemicals like flavonoids, lignans, xanthones, glycosides, tannins, triterpenoids and steroids and other phenolic compounds that protect against reactive oxygen species and DNA strand breaking [36,38]. The nanoparticles of *C. speciosus*, were described to have higher scavenging activities than the rhizome extract [39], and this is supportive to our study findings. They added that this might be attributed to the interaction between plant metabolites and metal ions during the nanoparticles’ formation which resulted in compounds with enhanced free radical scavenging properties [39]. The electrostatic attraction between phytochemicals that have negative charges and the nanoparticles that have neutral or positive charges synergizes and enhances the bioactivity [40].

STZ-induced DM was associated, in this study, with a significant decrease in serum testosterone which was in agreement with previous studies conducted on diabetic animal models [41,42]. Reduced testosterone level in DM might be attributed to a reduced number of Leydig cells and/or insulin deficiency which resulted in reduced production of follicle-stimulating hormone (FSH) and luteinizing hormone (LH) and consequently reduces testosterone production and fertility [43,44].

Reduced testosterone level may be behind the reduced prostate weight in diabetic rats that was evident in this study. The prostate of diabetic rats is affected by the lack of the anabolic activity of insulin and it is deprived of the regulatory mechanisms of testosterone which plays a basic role in the prostate gland development, epithelial proliferation and normal secretory function [45]. Reduced testosterone level and prostate weight suggest the inhibition of cell proliferation and increased apoptosis in the prostate as was previously described by [46], and this was evident in this study when assessing the prostatic immunoexpression of Ki67 and Bcl2 in diabetic rats, respectively. Treatment of diabetic rats with metformin and *C. speciosus* was associated with increased serum testosterone compared to the untreated diabetic rats, and this might be due to reduced blood glucose level that is inversely related to the testosterone level [47] as well as increased insulin [44].

*C. speciosus* extract was reported to reduce pregnancy among normal rats without inhibiting the sexual behavior of male rats, and this effect was not generated through the hormonal mechanism as the levels of testosterone, LH and FSH were not affected [48]. Although the findings of [48] appeared to be contradicted with what was observed in our study, there was a big difference in the two study designs as we have studied the effect of *C. speciosus* on testosterone in diabetic rats which was reduced due to decreased insulin. Therefore, enhanced insulin secretion by *C. speciosus* could significantly increase testosterone level. In this study, increased body weight was observed among diabetic rats treated with *C. speciosus.* In accordance with that, rabbits treated with *C. speciosus* extract after receiving hyperlipidemia-induced diet showed increased body weight [16]. This might be attributed to the high protein content in *C. speciosus* [49].

The free oxygen radicals and advanced glycation products induced by hyperglycemia were reported to enhance the production of inflammatory cytokines including TNF-α [50,51]. In this study, DM-associated inflammatory process was documented as gene expression of IL-6 IL-1β and TNF-α was significantly up-regulated in untreated diabetic rats, while it was significantly down-regulated after treatment with metformin- and *C. speciosus*. The latter is among the plants that have potent anti-inflammatory effects as it reduces the pro-inflammatory cytokines; TNF-α, IL-6, inducible NO synthase (NOS), and cyclooxygenase (COX-2) in many in vitro and in vivo studies [52] as well as in a clinical trial [53].

The ventral prostate of untreated diabetic rats showed enlarged, widely separated acini lined by thinner less folded acinar epithelium with many degenerated cells. These findings were in agreement with some previous studies [54,55]. In accordance with what we have observed in this study, the ventral prostate of control rats showed primary localization of Bcl-2 expression in the epithelial acinar cells using in situ mRNA hybridization [56]. It was reported that the relative abundance of Bcl-2 protein correlates with the survival of prostatic epithelial cells and is down-regulated with apoptosis [57]. This supported the findings of our study and those of [58] who reported a significant reduction in Bcl-2 expression in prostate of STZ-induced diabetic rats. Increased apoptosis in the ventral prostate in DM was proposed to be linked potentially to reduced testosterone level [59] which was confirmed in this study. Not only that, increased oxidative stress and reduced level of the antioxidant enzymes in DM directly induce apoptosis by damaging the DNA [60].

Ki67 immunoexpression was also significantly reduced, in this study, in the ventral prostate of untreated diabetic rats and this was in agreement with the finding of [61]. It was reported that prostatic involution that occur in experimental diabetes requires an imbalance in cell proliferation and death [61]. In support with that, this study revealed a reduction in cell proliferation and an increase in the cell apoptosis in diabetic prostate evident by reduction in the anti-apoptotic Bcl-2 expression. Although no previous studies were found to investigate the effect of *C. speciosus* or its nanoparticles on the structure of prostate in diabetic models, the study conducted by [19,35] revealed that both crude extract of costus and nanocostus could ameliorate STZ-induced histopathological changes in liver and pancreas with the superior effect of nanocostus. It was reported that the superiority of the nanostructure over the crude extract of costus concerning the antidiabetic effect, as well as the protective effect against diabetes-induced testicular damage, may be attributed to improved bioavailability and absorption of the nanoformula [14]. It was reported that the anti-apoptotic role of testosterone may contribute to the ability of testosterone to up-regulate the expression of anti-apoptotic genes such as Bcl-2 [62]. Therefore, increased testosterone in *C. speciosus*-treated diabetic rats was behind preserved prostatic structure, increased prostatic Bcl-2 and Ki67expression.

## 5. Conclusions

The protective effect induced by *C. speciosus* on the structure of the prostate of diabetic rats is evident in this study and might be directly mediated through down-regulating the expression of inflammatory cytokines and up-regulating antioxidant activity and indirectly mediated through the anti-hyperglycemic effect induced by enhancing insulin secretion. Investigations in this study were limited to the confirmation of the efficacy of the *C. speciosus* nanoparticles and exploring one possible mechanism behind this effect, although more mechanisms could be discovered in coming works. Further research for testing this effect in diabetic male patients is recommended.

## Figures and Tables

**Table 1:** The composition of *C. speciosus* Extract Loaded Nanostructured Lipid Carriers (CSE-NLCs).

Component	Amount
Oleic acid	500 mg
Compritol 888 ATO	150 mg
Precirol ATO5	350 mg
Dried plant extract	60 mg
PluronicF-68 1%	200 mg
Tween^®^80 1%	200 mg

**Table 2:** Summary of characterization of *C. speciosus* Extract Loaded Nanostructured Lipid Carriers (CSE-NLCs).

Characterization	Value
Particle size (nm)	568.4 ± 0.53
Polydispersity index	0.498 ± 0.06
Zetapotential mV	−39.00 ± 2.30
Encapsulation efficiency %	89.00 ± 1.90
Loading capacity %	4.37 ± 0.05

**Table 3:** Effect of *C. speciosus* on the blood glucose level (BGL), insulin, oxidants/antioxidant profile and testosterone in the serum of the studied groups.

	Control	*C. speciosus-*Treated	Untreated Diabetic	Metformin-Treated Diabetic	*C. speciosus-*Treated Diabetic
**Body weight (g) at the start of the experiment**	209.13 ± 5.63	208.33 ± 5.91 *p* = 0.92	211.50 ± 6.97 p1 = 0.94	210.96 ± 7.32 p1 = 0.84	214.04 ± 6.45 p1 = 0.89 p2 = 0.87
**Body weight (g) at the end of the experiment**	314.83 ± 43.24	304.83 ± 23.35 *p* = 0.95	261.33 ± 20.78 p1 = 0.01	296.33 ± 12.39 p1 = 0.24	299.17 ± 13.93 p1 = 0.16 p2 = 0.94
**Prostate weight (g)**	1.18 ± 0.07	1.17.00 ± 0.37 *p* = 0.89	0.78 ± 0.15 p1 = 0.02	0.96 ± 0.13 p1 = 0.84	0.90 ± 0.19 p1 = 0.65 p2 = 0.76
**BGL level (mg/dL) at the start of the experiment**	79.57 ± 7.81	67.50 ± 30.00 *p* = 0.95	81.24 ± 12.53 p1 = 0.93	84.17 ± 13.58 p1 = 0.89	79.00 ± 11.49 p1 = 0.91 p2 = 0.88
**BGL level (mg/dL) at the end of the experiment**	80.90 ± 5.39	88.45 ± 10.26 *p* = 0.98	354.07 ± 80.39 p1 < 0.001	259.85 ± 39.37 p1 = 0.01	268.90 ± 45.26 p1 = 0.03 p2 = 0.91
**Insulin Level in serum (µIU/mL)**	5.23 ± 0.40	5.30 ± 0.37 *p* = 0.92	2.28 ± 0.77 p1 < 0.001	3.56 ± 0.39 p1 = 0.003	3.42 ± 0.57 p1 = 0.01 p2 = 0.89
**Testosterone (ng/mL)**	28.69 ± 1.41	29.07 ± 2.64 *p* = 0.92	18.75 ± 2,11 p1 < 0.001	24.19 ± 3.76 p1 = 0.02	23.64 ± 2.88 p1 = 0.04 p2 = 0.93
**MDA in serum (nmoL/mL)**	1.27 ± 0.13	1.34 ±0.16 *p* = 0.93	2.20 ± 0.73 p1 = 0.004	1.47 ± 0.43 p1 = 0.04	1.37 ±0.18 p1 = 0.01 p2 = 0.93
**SOD in serum** **(µ/mL)**	19.32 ± 1.97	18.73 ± 3.42 *p* = 0.92	10.52 ± 3.00 p1 < 0.001	16.46 ± 3.18 p1 = 0.01	17.52 ± 2.35 p1 = 0.002 p2 = 0.89
**GPX in serum** **(µ/mL)**	54.49 ± 6.94	58.94 ± 9.89 *p* = 0.88	35.01 ± 5.87 p1 = 0.003	50.94 ± 5.41 p1 = 0.02	52.09 ± 10.92 p1 = 0.01 p2 = 0.91
**CAT in serum (µ/L)**	0.44 ± 0.08	0.47 ± 0.07 *p* = 0.95	0.16 ± 0.06 p1 < 0.001	0.36 ± 0.08 p1 = 0.001	0.39 ± 0.08 p1 < 0.001 p2 = 0.92

ANOVA test was used to compare the studied groups followed by Bonferroni*post hoc* test. Results are presented as mean ± standard deviation (SD). Significance was considered at *p* < 0.05. P, significance versus the control group. P1, significance versus the untreated diabetic group. P2, significance versus the metformin-treated diabetic group.

## Data Availability

The raw data included in this study will be made available by the corresponding author upon reasonable request.

## Supplementary Materials

Table S1: Gene specific primers utilized in this study.
