# Supplementary material for: Nanoparticles of Costus speciosus Ameliorate Diabetes-Induced Structural Changes in Rat Prostate through Mediating the Pro-Inflammatory Cytokines IL 6, IL1β and TNF-α
Source: Molecules. 2022 Feb 2;27(3):1027. doi: 10.3390/molecules27031027 (PMC8839105; doi:10.3390/molecules27031027)
Supplement: Supplementary file 1 [file molecules-27-01027-s001.zip › molecules-1563778-supplementary.pdf]

Table S1. Gene specific primers utilized in this study.

Gene	Sequence
rat-GAPDH	(Forward 5'-CAACTCCCTCAAGATTGTCAGCAA-3', Reverse5-'GGCATGGACTGTGGTCATGA-3'.
rat-TNF-α	(Forward 5'-CCCTGGTACTAACTCCCAGAAA-3', Reverse, 5'-TGTATGA-GAGGGACGGAACC-3'.
rat-IL-6	(Forward 5'-CTGCAAGAGACTTCCATCCAG-3', Reverse, 5'-AGTGG-TATAGACAGGTCTGTTGG-3'.
rat-IL1β	Forward 5′-CTTATGCCTTCAACGGAGGT-3, Reverse 5′-GTGTCTGAA-GCAACGATGGA-3′.
